# A new trial monitoring plan (TMP) template for clinical trials: output from a Delphi process

**DOI:** 10.1186/s13063-024-08601-z

**Published:** 2024-11-09

**Authors:** Shiva Taheri, Victoria Yorke-Edwards, Matthew R. Sydes, Talia Isaacs, Sharon B. Love

**Affiliations:** 1 MRC Clinical Trials Unit at UCL, 90 High Holborn, London, WC1V 6LJ UK; 2 Centre for Advanced Research Computing, University College London, 90 High Holborn, London, WC1V 6LJ UK; 3 MRC Clinical Trials Unit at UCL, University College London, 90 High Holborn, London, WC1V 6LJ UK; 4 Data for R&D, NHS England, London, UK; 5 Faculty of Education and Society, IOE, UCL’s, University College London , 20 Bedford Way, London, WC1H 0AL UK

**Keywords:** Trial monitoring plan, Clinical trial monitoring, Trial conduct, Delphi survey, Consensus

## Abstract

**Background:**

Monitoring is a crucial part of trial conduct and ensures that participants’ data is fairly represented, and future healthcare information is enhanced. This project aims to improve trial monitoring by creating a trial monitoring plan (TMP) template with input from individuals experienced in monitoring clinical trials.

**Methods:**

A review of monitoring plans received from UK Clinical Research Collaboration (UKCRC) registered clinical trials units (CTU)s created the basis for a preliminary TMP template and a Delphi survey. The TMP template was finalised after 2 rounds of a Delphi survey and a two-part consensus meeting including trialists with experience and expertise in monitoring clinical trials.

**Results:**

We received 31 monitoring plans from UKCRC-registered CTUs and reviewed over 800 monitoring items for inclusion in a TMP template, selecting items based on guidelines such as Good Clinical Practice (GCP) and our monitoring experiences. For certain items, further expert input was required. A total of 66 items were chosen for a Delphi survey involving 47 participants from 25 UK CTUs and industry. After the first round, all 66 items were retained, and six additional items were suggested by Delphi participants. In the second round, 37 items reached consensus for inclusion in the TMP template. The Delphi process was followed by a consensus meeting with 9 participants from 9 UK CTUs and industry. Participants in the consensus meeting voted on the 32 further items that had not reached the definition of consensus within the Delphi, regarding each item’s inclusion in or exclusion from the TMP template. The voting resulted in 18 items being excluded, leaving 14 items to be included in the TMP template. The process overall resulted in a standardised TMP template with input from many individuals with interest, experience, or expertise in monitoring clinical trials.

**Conclusion:**

A TMP template was developed building on the currently used monitoring plans and with input from those experienced in clinical trial monitoring. Using a centrally developed good quality TMP template should contribute towards maintaining consistency in monitoring standards across all CTUs, resulting in higher research quality and improved quality assurance. Its use should provide reassurance to participants that their participation is carefully monitored to ensure that their data or any samples provided are treated with confidentiality, integrity, and respect and that their rights and well-being are protected.

**Supplementary Information:**

The online version contains supplementary material available at 10.1186/s13063-024-08601-z.

## Introduction

Research is a vital pillar in leading healthcare systems. A good quality clinical trial answers question(s) to improve health and care. The quality of a clinical trial depends on the adoption of an effective quality control process that ensures participant safety and rights and that trial and data integrity are always protected [1]. This process is called monitoring. Monitoring is important as it brings quality assurance to the conduct of research; it also mitigates risk and detects issues at an early stage when remedial action can reduce further issues [2].

Monitoring can be conducted on-site, centrally, or remotely. According to Good Clinical Practice (GCP) guidelines [1], on-site monitoring involves conducting visits at the physical location of where the clinical trial is being conducted. Centralised monitoring refers to the remote evaluation of data collected from trial sites, while remote monitoring oversees trial activities and assesses data from a distance, without on-site visits.

A trial monitoring plan (TMP) specifies the planned and systematic actions devised to ensure that the research is designed, conducted, documented, and reported in compliance with Good Clinical Practice (GCP), research governance frameworks [3, 4], and any other applicable regulatory requirements [1, 5]. TMPs are written to reflect the research’s risk level, and patient population, and are reviewed as needed, for example, in the event of a protocol amendment or any changes to the trial’s risk assessment, to ensure that they are up to date. The purpose of a TMP is to document procedures for monitoring and action plans before, during, and at the end of the research [1, 6]. These plans may include information on on-site, centralised and remote monitoring activities, and details of the research parameters to be monitored [6]. This includes, for example, when the first monitoring visit should be conducted and the frequency of any monitoring activity thereafter. A TMP is specific to each trial, and it is created during the initial stages of the trial set-up to help with conduct planning. Creating this document at the initial stages of the trial helps with ensuring the protocol is effective, assessing risks and planning mitigation strategies before the trial begins. A TMP can also be created or revised prior to the start of recruitment to ensure the plan reflects the trial’s current risk profile.

Currently, in the UK, each clinical trials unit (CTU) develops its own TMP based on its specific regulations and standard operating procedures (SOPs), ICH GCP guidelines [1], and regulatory settings guidelines, e.g. UK Medicine and Healthcare products Regulatory Agency (MHRA) [7] and U.S. Food and Drug Administration (FDA) [8], and other relevant guidelines [6]. There is considerable variation in how CTUs create their monitoring plans, essentially duplicating effort every time this process is repeated. Creating a TMP is a time-consuming task that demands expertise and knowledge. Moreover, when monitors transition between, or work across CTUs, they must often adapt to different formats of monitoring plans to present essentially the same information. While some CTUs benefit from having more experienced staff who can produce well-structured monitoring plans, others have lacked similar resources and expertise. This highlights the necessity for a standardised TMP template accessible to all, ensuring widespread benefits across all CTUs.

The MHRA emphasises risk-appropriate monitoring in clinical trials to reduce unnecessary burdens while ensuring critical aspects of clinical trials are closely monitored to protect participant safety and ensure data reliability [7]. This increased focus on monitoring by the MHRA indicates a commitment to enhancing the quality and safety of clinical trials, ultimately benefiting both participants and the overall integrity of the trials. Recent research has been conducted to offer checklists and templates for enhancing clinical trials protocols and statistical analysis plans [9, 10]. Attention has also been directed towards improving recruitment and retention in clinical trials. This is therefore an appropriate time for this study’s aim to develop a comprehensive trial monitoring plan template to uplift the overall standard of monitoring in clinical trials, which can be used for both CTIMP (Clinical Trials of Investigational Medicinal Products) and non-CTIMP (Trials that do not involve an Investigational Medicinal Product (IMP)) trials across different trial phases.

## Methods

### Research design

The data collection process began by gathering TMPs from UK Clinical Research Collaboration (UKCRC) registered CTUs. A comprehensive list of items that could be included in the TMP template was created. This list was compiled by reviewing all the monitoring plans collected from CTUs and underwent an iterative review process. This list formed the basis of a Delphi survey and a consensus meeting for individuals with monitoring experience or expertise to express their opinion of the need for the items to be included in a TMP template.

### Collecting monitoring plans from all CTUs

In October 2022, an email explaining the aim of the project was sent to the monitoring leads of all 52 UKCRC registered CTUs, via the UKCRC network. There is much variation in the monitoring terminology across the clinical trial community, so the email explained what a trial monitoring plan is, acknowledging that some units may refer to it as a ‘risk assessment plan’ or a ‘data monitoring plan’. The email asked CTUs to share their current monitoring plan documents with the research team.

### Data extraction and classification

Wording items were extracted from the received monitoring plans and compiled into an Excel spreadsheet by the lead author. They were listed individually and grouped under sections such as *‘study details’*, *‘introduction to the trial’*, and *‘Adverse Events and Serious Adverse Events’*, which were found across the CTU monitoring plans and therefore were determined should be included in a TMP template. Such sections of items were similar in many CTU monitoring plans with some having more sections than others.

Duplicate items were identified, and variation in wording was taken into consideration. Multiple phrasings of the same items were entered into the spreadsheet to capture different wordings from all the monitoring plans. Optimal wording for the final TMP template would be ascertained using the Delphi survey.

Table 1 presents the collective count of CTUs that included an item referring to the *‘recruitment target’*. Various CTUs used different expressions to represent this item. After reviewing all the CTUs’ monitoring plans, the phrase *‘overall recruitment target’*—the most frequently adopted wording amongst the CTUs—was selected.

**Table 1:** Example of items found in 31 CTU monitoring plans with different wordings with the same meaning

Item wording	Number of CTUs including this item wording
Sample size	3
Overall target recruitment	3
Overall recruitment target	6
Number of participants	4

Two authors with considerable monitoring experience (ST, SBL) independently reviewed every item and classified each item into one of three categories: items to be included *‘directly in the TMP template’*, items to be included *‘in the Delphi’*, or items *‘not included at all’*. This classification was made by looking at the number of monitoring plans that included each item and the importance of the item based on the monitoring experience of each author. Both authors discussed items where there was no immediate agreement and mutually decided whether to include them directly in the TMP template or Delphi or not to include them at all. When encountering an item on which authors’ opinions greatly diverged or when it was felt important to seek the input of other experts, those items were added to the Delphi list.

Those items that were classified to be directly added to the TMP template were those found in the majority of CTU monitoring plans. Additionally, the FDA [8] and the MHRA [7] guidelines were taken into consideration. For instance, *randomisation procedure (for confirming that randomisation is performed according to the protocol and investigational plan)* was an item that was included in the TMP template based on recently published FDA guidelines [8].

Any items that did not appear in at least 3 CTU monitoring plans and seemed less significant were not included at all. Careful consideration was given to these items before deciding not to include them. Considerations included whether these items exist within the trial protocol and how readily accessible they are. The overarching aim was to maintain an alignment between the monitor’s duty to check the contents of the protocol and ensuring that the TMP template contained all the necessary information without making it unnecessarily detailed.

The preliminary TMP template and list of items for the Delphi survey were reviewed extensively over a few months. The final list of items for round 1 (Supplementary File 1) included a comprehensive list of 66 items that could be included in the TMP template.

## The Delphi survey

### The Delphi survey invitations

The Delphi survey was used as a tool to reach consensus amongst individuals with monitoring experience, expertise, and interest primarily in the UK. An email was sent via the UKCRC registered CTU network on 27 April 2023 inviting everyone to participate, followed by an email on 18 May 2023. The email contained the link to the Delphi survey, the participant information sheet (PIS), and the draft trial monitoring plan template. Participants were fully informed of the number and timing of the Delphi rounds and the commitments that were required to participate. Weekly Tweets on Twitter (now ‘X’) were also posted by the MRC clinical trials unit at UCL (MRC CTU at UCL) official account to inform the monitoring community about the survey. Additionally, individual emails were sent to the CTUs that had shared their monitoring plans. Contacts were made with the Medicine and Healthcare products Regulatory Agency (MHRA) and authors of *‘Guidelines for the Content of Statistical Analysis Plans in Clinical Trials’* [10]. Industry contacts also received individual emails to encourage their input in the survey. Ethics approval was obtained from the UCL Research Ethics Committee (REC) for the study. Consent to take part was indicated through survey participation.

### Delphi software and rounds

An online software program called ‘Comet Initiative Delphi Manager’ [11] that has been used in a number of previous clinical trial methodology research studies [10, 12] was purchased from the University of Liverpool. The first round of the Delphi survey started on 27 April 2023 and lasted 5 weeks. In round 1, the Delphi participants were able to comment on the wording of the Delphi items and could suggest additional items to be included in the template. There was a 2-week interval between round 1 and round 2. All individuals who participated in round 1 were invited to complete round 2, and new participants were also encouraged to join. Announcements about the opening of round 2 were made via the MRC CTU at UCL Twitter account. Round 2 started on 21 June 2023 and lasted 3 weeks. Weekly reminders were sent to participants to complete the survey.

### Scoring process

Participants in each round were asked to score the importance of each item to be included in the final TMP template. They were provided with a copy of the proposed draft TMP template to determine where the Delphi items were situated in the template. Participants were informed that they were choosing items to be included in a TMP template that could be subsequently tailored to the specific needs of a clinical trials unit and of individual trials. Thus, where the presence of an item was not relevant to some types of trials, it could be removed when using the template for that specific type of trial. A 9-point Likert scale for scoring was used. The response options were presented with scores 1–3 labelled as *‘not important’*, 4–6 labelled as *‘important but not critical’*, and 7–9 labelled as *‘critical’*, and 10 labelled as *‘unable to respond’*. For each item in round 2, previous participants’ responses were presented in a graph showing the total number of participants who had rated that item in round 1 along with the percentage of participants who chose each score. Additionally, participants were shown their own score in round 1 and given the option to either revise their own answers or maintain them unchanged [13].

### Consensus definition

A modified Delphi approach was used, involving a structured questionnaire instead of the classic Delphi approach of open-ended questions [14]. This method was chosen because the issue in this research—lack of a standardised TMP template—had already been identified, and structured statements were generated by reviewing 31 CTU templates. The classic Delphi method can result in a large number of statements, leading to lengthy rounds and a time burden for participants [14]. In this case, using the classic method would have been impractical due to the approximately 800 items derived from the 31 CTU TMPs reviewed, making the modified method more appropriate.

Consensus was predefined for this study [10, 12], as items that were rated critical by 70% or more of the participants and not important by less than 15% of the participants were determined to have reached consensus for inclusion in the TMP template [10, 12]. Conversely, items that were rated not important by 70% or more of the participants and critical by less than 15% of the participants were determined to have reached consensus for exclusion from the TMP template. This definition has been used in other Delphi surveys conducted for similar research and was deemed appropriate by the authors [10, 12]. Furthermore, a median of 7–9 was considered to mean agreement on individual items, as suggested by Trevelyan et al. [14], and an interquartile range (IQR) ≤ 2 was considered indicative of consensus across all items, as suggested by Gracht et al. [15].

### Consensus meeting

Invitations to join the consensus meeting were extended to participants who had shown interest in participating in the Delphi survey. The consensus meeting participants were presented with the TMP template, the Delphi results, and information on where the Delphi survey items were situated in the current template. The participants were shown the result of the Delphi for each question. The results were presented in graphs which showed the number of participants that rated each item in round 1 and round 2 and the total response in each category of the Likert scale [1–10]. Participants were given the opportunity to discuss each item and ask questions before votes were taken on each item as to whether they were to be included in or excluded from the TMP template.

## Results

A total of 31 monitoring plans were received from 52 UKCRC registered UCTs, 20 after the first email, 11 after a follow-up email. One further unit expressed interest in the study but had a monitoring plan known to be inadequate due to a recent inspection; they were working on improvements and chose not to share it yet. Another CTU explained they outsource monitoring to a Clinical Research Organisation (CRO) and do not have a designated plan. The remaining 19 CTUs did not reply. The full list of UKCRC registered CTUs at the time this study was done, highlighting those CTUs that shared their monitoring plans, can be found in Supplementary File 2.

The spreadsheet containing items extracted from the 31 monitoring plans contained over 800 items. Round 1 of Delphi survey consisted of 66 items. Approximately 10% (7/66) of the items that were included in the round 1 were ones where authors did not reach mutual agreement on when reviewing and classifying each item’s category. Six additional items were suggested by participants in round 1 (see Supplementary File 3). These additional items were reviewed by the authors for suitability and duplication and added to round 2. The authors decided to not omit any items from round 2 that were included in round 1, to be able to have a full analysis of the data [14]. Therefore, round 2 included all items from round 1 as well as additional items suggested by participants in round 1, making a total of 72 items in round 2.

### Delphi participants

**Fig. 1:**
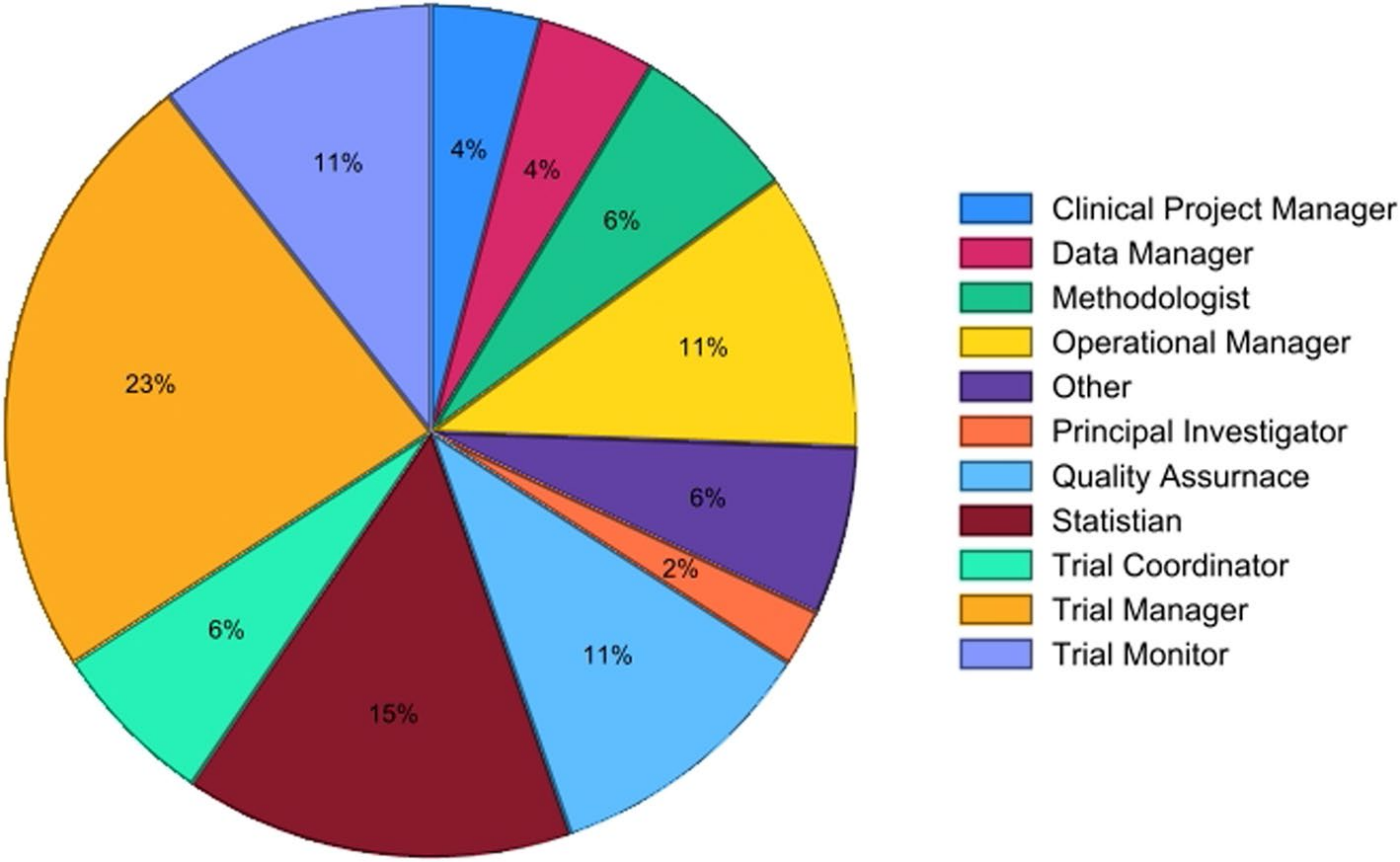
Role distribution of all 47 Delphi survey participants

Demographic data was collected at the time of participants’ registration for the Delphi survey. The 47 Delphi participants across both rounds were from 25 different UKCRC registered CTUs and industry. The participants held various roles within the clinical trials field. Distribution of roles can be seen in Fig. 1.

The participant demographics in round 2 closely resembled those in round 1, except for the inclusion of three new participants: one clinical trial monitor, one quality assurance manager, and one trial manager.

Of the 47 participants in round 1, 43 (91%) fully completed the survey, while 4 (9%) participants partially completed the survey. In round 2, 37 (79%) participants fully completed the survey, while 3 (6%) partially completed the survey, and 7 (15%) round 1 participants did not engage in round 2. Additionally, round 2 was open to anyone who was interested in taking part in the survey, regardless of whether they participated in round 1. Therefore, in addition to the 37 participants who fully completed round 2, there were 3 new participants, making a total of 40 (93%) participants who fully completed round 2, and 3 (7%) participants who partially completed round 2.

**Fig. 2:**
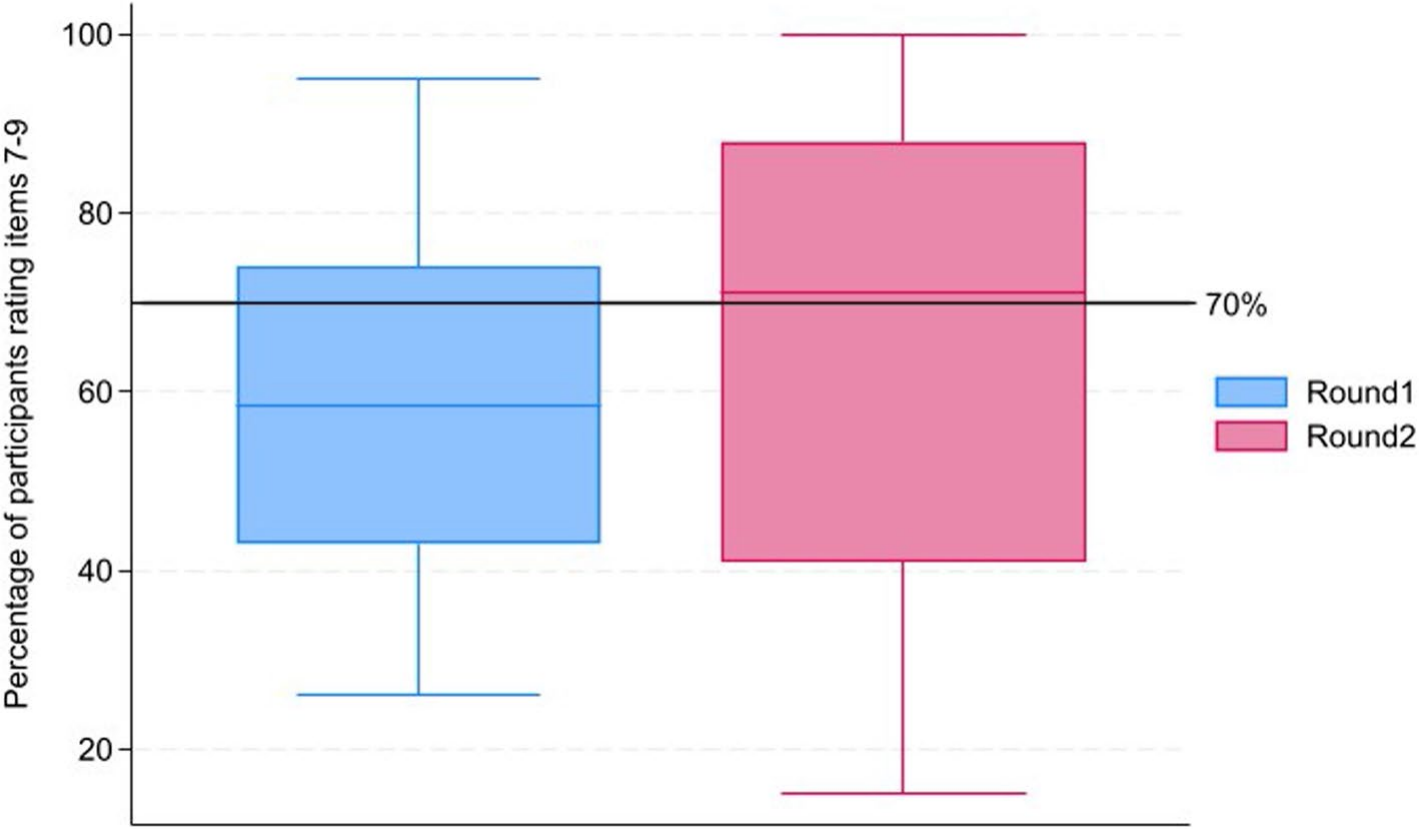
Distribution of the percentage of participants rating items with a Likert score of 7–9 (critical) in round 1 vs. round 2. The black line represents the threshold where 70% or more of participants rated items as critical

On completion of both Delphi rounds, the number of items for which more than 70% of the participants responded with a Likert score of 7–9 (critical) increased by 18%, from 22/66 (33%) in round 1 to 37/72 (51%) in round 2 (Fig. 2). Additionally, median scores were compared as suggested by Trevelyan [14] to determine if agreement was reached amongst participants on individual items. Comparing the items’ median score, in round 1, 47/66 (71%) items had a median score of 7–9, and in round 2, 51/72 (71%) items had a median score of 7–9. There were no items for which 70% or more of the participants responded with a Likert scale of 1–3 rated *‘not important’* in either round.

**Fig. 3:**
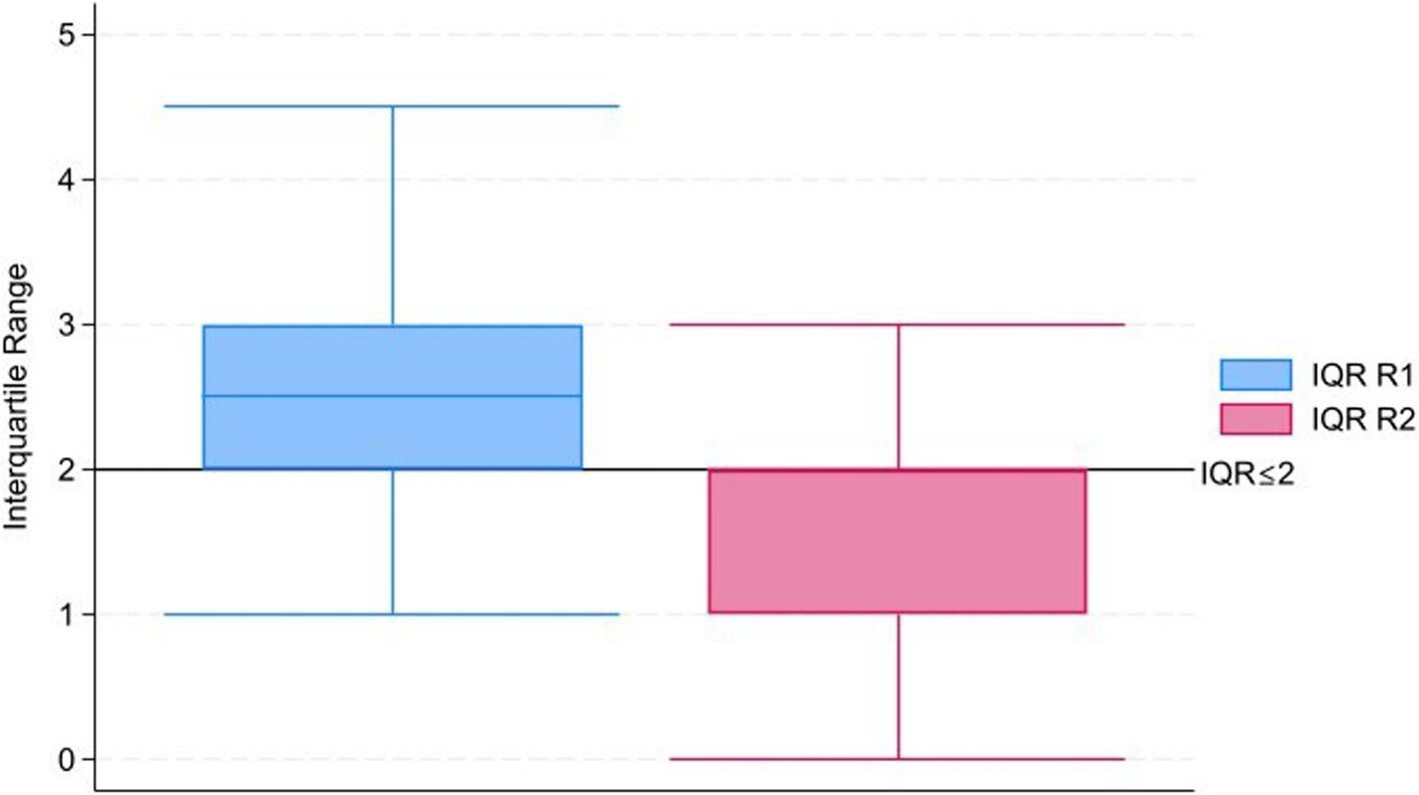
Comparing the interquartile range in items in round 1 vs. round 2. The black line indicates where the interquartile range is ≤ 2

Furthermore, considering the interquartile range (IQR) ≤ 2 as suggested by Gracht et al. [15] as an indication of consensus across all items, the number of items with an IQR ≤ 2 in round 1 is 29, and in round 2, it is 56, which shows that the variance of response is reducing across the group of items (Fig. 3) [15]. Additionally, the number of comments in round 2 was lower than that in round 1, which is another indication of group opinion moving towards consensus [16].

The results of the Delphi study showed that the items with the highest number of participants *‘unable to respond’* were ‘*checks for serious adverse events for medical devices’*, *‘use of metrics in monitoring clinical trials’*, and* ‘trial oversight of vendor’*. These items were discussed in more details at the consensus meeting. After round 2, the results and analysis were reviewed by the authors. It was concluded that with 79% of the items (86% of the original list of items in round 1) likely to stay in their critical categories (i.e. had been scored 7–9 by more than 70% of the participants), it was justifiable to not have another survey round as it was clear that the group was moving towards consensus. Additionally, to avoid participants attrition and fatigue [14, 15], it was decided to stop the Delphi survey at 2 rounds and move on to the consensus meeting where the results would be discussed, and participants would have a final opportunity to influence the development of the TMP template.

### Consensus meeting

**Fig. 4:**
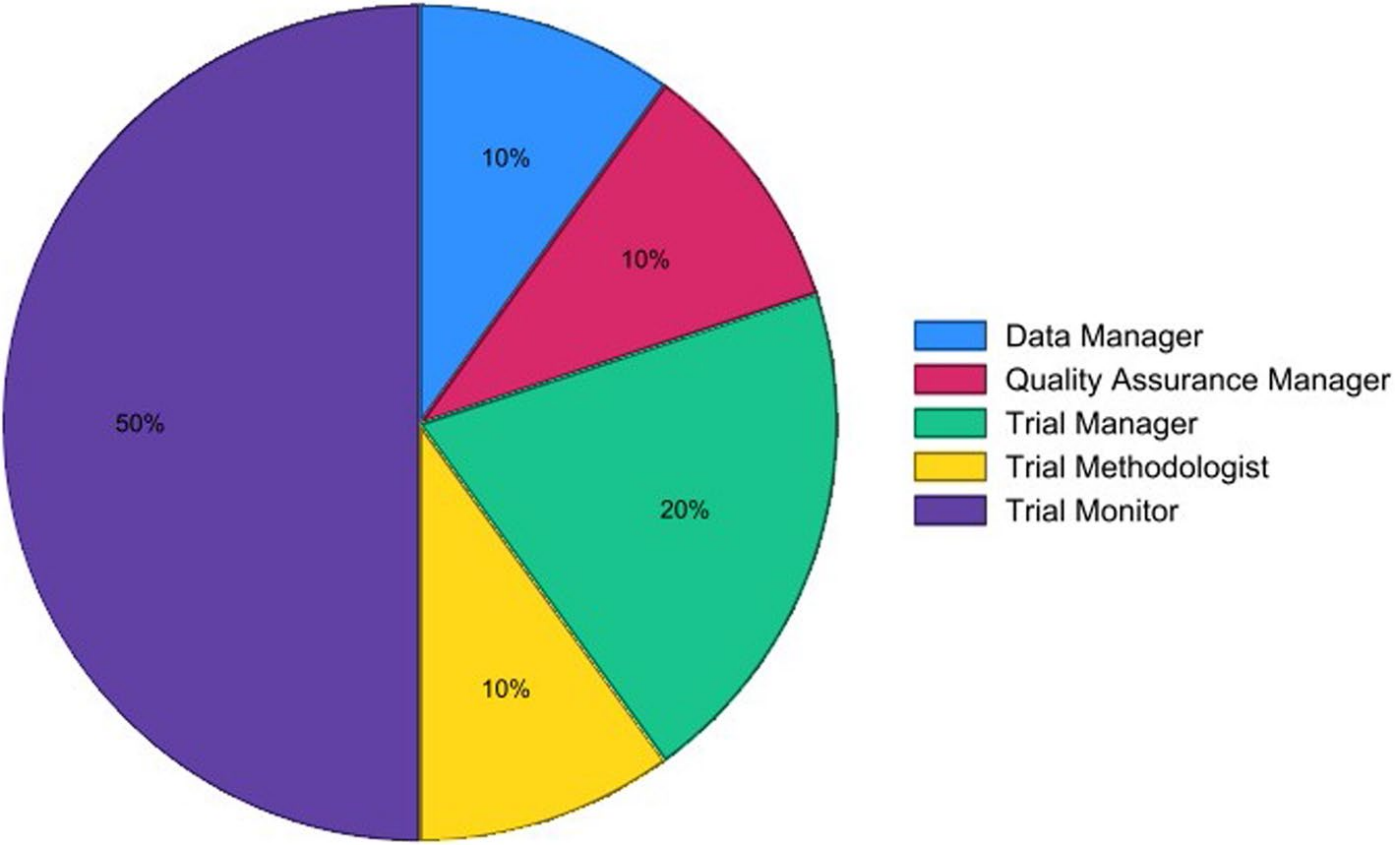
Role distribution of consensus meeting participants, consisting of 10 different people across the two sessions

The consensus meeting took place in two half-day sessions on 6 September 2023 with 9 participants and on 8 September 2023 with 8 participants. The participants were from 9 UK CTUs and industry. Distributions of participants across various roles in clinical trials can be seen in Fig. 4.

At the meeting, 37 items that had reached consensus during the Delphi survey were presented, and participants were asked to make any comments, including the wording of the items or to voice any objections to their inclusion in the template. Additionally, 6 items that had been suggested by participants in round 1 were presented separately to emphasise that they had been rated in only one round (round 2). Of the 6 additional items, 3 had reached consensus for inclusion during round 2 (as part of the 37 items presented), and participants needed to vote on the other 3 items that had not. After discussion, participants in the consensus meeting voted on the 32 further items that had not reached the definition of consensus within the Delphi, regarding each item’s inclusion in or exclusion from the trial monitoring plan template. The voting resulted in 18 items being excluded, leaving 14 items to be included in the TMP template. Participants engaged in discussions regarding the inclusion of items in the TMP template considered factors such as their significance to trial monitoring, the ease of locating the information within the protocol, and whether these items were better suited for inclusion in the monitoring template or other trial-related documents, such as trial SOPs, data monitoring plans, or other working instructions. The authors conveyed to meeting participants that this template could be used in its current form when presented to the CTUs or that it could be customised to align with the specific standards and requirements of each CTU. It was also clarified that while some CTUs have a comprehensive TMP template, others may not. Consequently, participants were encouraged to consider this when voting for items.

The template was finalised based on the discussions held during in the consensus meeting. The template contains a comprehensive list of items that should be included in a trial monitoring plan template (Supplementary File 4).

## Discussion

Trial monitoring plans from various CTUs were gathered, including those with well-established documentation and those requiring improvement. This selection provided us with a diverse sample of monitoring plans. The TMP template was developed using transparent methods and involving many stakeholders with diverse expertise in monitoring clinical trials.

In the Delphi survey, no items were rated as *‘not important’* by 70% or more of the participants in either round. However, 18 items were voted for exclusion from the TMP template during the consensus meeting, where items were discussed at length and collective decisions were made about the benefits of excluding them. This highlighted the advantage of having a consensus meeting in being able to have detailed discussions of participants’ varying perspectives.

Items that had a high percentage of ‘*unable to respond’* were discussed in detail during the consensus meeting. Of these, *‘checks for serious adverse events for medical devices’* and *‘use of metrics in monitoring clinical trials’* were voted to be included in the TMP template by the consensus meeting participants. Additionally, *‘trial oversight of vendor’*, which also had a high number of *‘unable to respond’* score, was discussed at length. In this instance, it was decided that having specific items on vendor oversight could make the template overly complicated as some CTUs may not have the staff with the expertise to monitor external vendors. This item was therefore modified to a prompt for the trial monitor to refer to the *‘vendor oversight SOP’* at the specific CTU.

There was considerable interest in this study within the UK monitoring community, as shown by the participation of most CTUs (31/52) that shared their monitoring plans and the Delphi survey, which involved participants from 25 CTUs across the UK. Additionally, many CTUs expressed interest in learning more about the study and requested presentations.

The new TMP template will be tested and validated to ensure it aligns with its intended purpose. This will be done by piloting the template with CTUs that have the capacity to do so. The piloting process involves using the new TMP template in a trial for at least 6–9 months. The pilot phase at each CTU will begin with a qualitative interview to capture initial experiences of using the new template, followed by another interview at the end of the piloting period. Qualitative analysis of the interviews will be conducted to evaluate the efficacy of the template in an evidence-based manner, with updates made to the template if required.

The new template will be made publicly available via open access repository Zenodo, thereby extending accessibility within the global clinical trials community. Trial sponsors and their research teams will be encouraged to use it. CTUs can adapt the template to their own unique needs or to make it compliant with their SOPs and other working instructions. The TMP template also includes instructions on how to use it and emphasises that it is not a standalone document; rather, it should be used in conjunction with the CTU’s SOPs, trial protocol, or any other documents related to the conduct of the trial or required by the sponsor. The template can be used for both CTIMP and non-CTIMP trials across different trial phases.

There are some limitations to our research. Although we received 31 monitoring plans from UK CTUs, we recognise that 19 CTUs did not respond. Amongst the 19 non-responding CTUs were some larger units with extensive trial portfolios. However, the 31 UK CTUs that participated also included units with large trial portfolios, covering both CTIMP and non-CTIMP trials. While 31 CTUs may be considered small to represent the monitoring processes of UK clinical trials, it is important to note that the data is limited to UKCRC-registered CTUs, which have met the high standards required for UKCRC accreditation [17].

Additionally, there are also limitations in Delphi studies. While they aim to identify group consensus, this does not necessarily equate to the ‘best,’ ‘expert,’ or ‘correct’ result [14].

## Conclusion

Although regulators like the MHRA emphasise the importance of monitoring and there is a great interest in risk-based monitoring of clinical trials, there has still been a lack of clear guidance regarding the minimum contents of a trial monitoring plan template. As a result, there has been a wide range of variation in monitoring practices amongst the UK academic trial units [17]. This research study further highlighted this issue by reviewing 31 monitoring plans received from UK CTUs, which showed a wide range of differences between plans.

Utilising the TMP template can benefit healthcare systems and patients. It enables the completion of high-quality clinical trials that are evidence-based, efficient, and effective. CTUs can improve their monitoring practices and standardise the way monitoring is performed by implementing the TMP template. Furthermore, research participants can have confidence that their participation is being carefully monitored to ensure that their data or any samples provided are treated with confidentiality, integrity, and respect and that their rights and well-being are protected.

A good TMP template has the potential to improve the transparency and completeness of TMPs, which will benefit the investigators, trial participants, future patients, and the healthcare system.

## Supplementary Information

Supplementary Material 1: Final list of Delphi items round 1.

Supplementary Material 2: List of 52 Clinical Research Collaboration (UKCRC) registered clinical trials units (CTU)s, as of 28th November 2022.

Supplementary Material 3: Additional items recommended by participants during Delphi round 1.

Supplementary Material 4: Trial monitoring template.

## Data Availability

The dataset supporting the analysis and results of this article is available upon request. Please contact the corresponding author for more information.
