# Supplementary material for: A new trial monitoring plan (TMP) template for clinical trials: output from a Delphi process
Source: Trials. 2024 Nov 9;25:748. doi: 10.1186/s13063-024-08601-z (PMC11549859; doi:10.1186/s13063-024-08601-z)
Supplement: Supplementary file 1 — Supplementary Material 1: Final list of Delphi items round 1. [file 13063_2024_8601_MOESM1_ESM.docx]

## Supplementary File 1: Final list of Delphi items Round 1

| **1. Study details** |
| --- |
| Purpose |
| CTIMP/non-CTIMP |
| **2. Introduction to the trial: Summary of study design/Trial overview** |
| Overall recruitment target |
| Primary outcome measures |
| Secondary outcome measures |
| Duration of patient recruitment |
| Duration of follow up: (choose as appropriate)  Per patient  The trial |
| Intervention(s) |
| Is the trial placebo or standard of care controlled? |
| Describe any specific regulatory requirements: e.g., The intervention is/is not being used in a licensed indication OR The data from the trial will/will not be used to support a licensing application OR The trial is/is not supporting a license change. <include details of international regulation e.g., FDA, Medical Devices, or other specific regulations> |
| Describe other issues specific to the treatment under study |
| **3. Monitoring** |
| Who will monitor the study? e.g., Sponsor/sponsor delegate |
| What will be the first monitoring time-point? |
| **4. Central Monitoring Activities** |
| Review of IMP shipment and delivery documentation |
| Review of IMP dosage calculations |
| If medical device trial SADEs reported to manufacturer (if not delegated to CTU) |
| Out-of-hours emergency cover arrangements- Where participants are provided with out of hours contact details for site staff (e.g., on a Participant ID card or PIS) indicate how this will be verified (e.g., for high-risk trials a test procedure may be put in place). |
| **4.1 Data Checks** |
| Checks for missing or invalid data (range and consistency checks) |
| Hard-copy CRFs and patient completed questionnaires validation: e.g., Where the CRF is a hard-copy, the content of approximately 10% of case report forms (CRFs) and patient questionnaires entered at sites will be checked (or double entered) to ensure the accuracy of data input. **(An error rate of <3% will require no further action, however if the error rate is >3%, a 100% check of forms will be undertaken.)** |
| **4.2 Protocol Deviation** |
| Review of Visit Window Thresholds |
| Other- Specify (e.g., Central review of adherence to the protocol and plausibility of the data, review of any questionnaires for completeness, time of randomisation and intervention consistent with clinical context, expected variability in items such as age, disease severity etc.) |
| **5. On-Site Monitoring activities** |
| Checking understanding and adherence to study protocol, procedures, and governance requirements (including any conditions in regulatory or ethics approval) |
| Review Medical/study records and results of eligibility assessments for <X%> of participants to confirm participant eligibility |
| Verification that resources and facilities remain adequate |
| Verification of appropriate oversight and documented delegation by the local investigator |
| Is the Site Delegation Log the original of the latest copy filed in Site Master File? |
| Has the Site Visit Log been completed at each visit? |
| Availability of completed source documents and CRF for the monitoring visit. |
| Source document completion in accordance with the ALCOA principles check. |
| **5.1 Protocol Deviation and Compliance** |
| Verification of missing visits, examinations, or tests. |
| Verification of lab reports reviewed, signed, and dated appropriately. |
| Have protocol deviations been reported appropriately? |
| Have any new protocol deviations and/or regulatory or GCP deviations occurred at site since the last visit? |
| **5.2 Site Staff Discussion** |
| Discussions with site staff and review of site staff training requirements (current documents and training present, staff changes documented, CVs, GCP, delegation log). |
| Other- Add any additional checks to be performed during on-site monitoring visits for this trial. |
| **5.3 Documents and systems to be reviewed** |
| Completion of annual progress and safety reports (as appropriate) |
| Randomisation processes |
| Recruitment rates |
| Screen failure |
| Withdrawal rates |
| **6. Source Data Verification (SDV)** |
| Is any SDV to be performed? Yes/no |
| Which patients need SDV and how will you select them?  e.g., number/percentage of patients and how you will select them, first patient at each site. |
| What data needs SDV? e.g., eligibility, outcome data, or all data |
| Question for Delphi respondents: Do you want a prompt list of data to SDV on the template? |
| Describe what source data will be available as a hard copy, and what will be available electronically and how access arrangements will be set up. |
| **7. Routine Monitoring Visits** |
| Selection criteria for participants to be reviewed during Routine Monitoring Visits- This may be on request of the TMG or following review of central monitoring reports, (e.g., participants who have a high number of SAEs reported) or on a percentage of participants (e.g., 10% selected at random). |
| **8. Remote monitoring activities** |
| Ongoing training/motivation meetings and teleconferences. |
| Completion of annual progress and safety reports (as appropriate). |
| **9. Metrics** |
| Question for Delphi Respondents: Do you want to see a prompt list of metric examples on the template? |
| Or do you want to have the option to use your unit specific list of metrics? |
| **10. Site Initiation Visit** |
| List of trainings to occur during the site initiation visit |
| Request for submission of Trial Equipment calibration records |
| Confirmation of Critical Documentation held– both regulatory and trial/site-specific |
| Obtaining confirmation that the site staff have completed the trial-specific training and are made aware of the operational requirements |
| **11. Close out visit** |
| Definition of end of trial |
| The Investigator Site File must be reviewed and confirmed as complete by the Trial Manager, prior to archiving. |
| All outstanding payments must be reviewed and invoiced |
| **12. Pharmacy Monitoring (Ordering and Storage of IMP)** |
| Are there adequate stocks within expiry dates for the planned patients? |
| Are storage temperatures adequately monitored by pharmacy staff? |
| Have any temperature excursions occurred? |
| Have any temperature excursions been appropriately managed? |
| **13. Trial oversight of vendor** |
| Onsite vendor monitoring |
| Central monitoring of vendor duties |
| Review of vendor related deviations (if required) |
| Completion of Vendor status reports |
