# Supplementary material for: A new trial monitoring plan (TMP) template for clinical trials: output from a Delphi process
Source: Trials. 2024 Nov 9;25:748. doi: 10.1186/s13063-024-08601-z (PMC11549859; doi:10.1186/s13063-024-08601-z)
Supplement: Supplementary file 2 — Supplementary Material 2: List of 52 Clinical Research Collaboration (UKCRC) registered clinical trials units (CTU)s, as of 28th November 2022. [file 13063_2024_8601_MOESM2_ESM.docx]

**Supplementary File 2:** List of 52 Clinical Research Collaboration (UKCRC) registered Clinical Trials Units (CTU)s, as of 28^th^ November 2022

**Those highlighted shared their monitoring plans with the study team:**

Name of registered CTUs (Fully registered)

1. Barts CTU
2. Cambridge CTU
3. NHS blood and transplant CTU
4. Swansea CTU
5. CCTU at UCL
6. Royal Marsden CTU
7. Papworth CTU (Cambridge)
8. Surrey CTU
9. Centre of trials research (Cardiff)
10. Derby CTU
11. Glasgow CTU
12. Newcastle CTU
13. North Wales Organisation for Randomised Trials in Health (& Social Care) – NWORTH
14. Peninsula CTU
15. Priment CTU at UCL
16. Centre for Healthcare Randomised Trials (CHaRT) Aberdeen
17. Norwich CTU
18. Bristol CTU
19. NPEU CTU Oxford
20. Keele CTU
21. Intensive Care National Audit & Research Centre ICNARC (London)
22. Oxford Primary Care & Vaccines Collaborative CTU
23. CaCTUS
24. CRUK & UCL Cancer Trials Centre (London)
25. Cancer Research UK Clinical Trials Unit (CRCTU) Birmingham
26. Leeds CTRU
27. MRC CTU at UCL
28. Imperial CTU
29. Warwick CTU
30. Northern Ireland CTU
31. Leicester CTU
32. Sheffield CTU
33. Southampton CTU
34. ICR-CTSU Sutton
35. Liverpool Trials Collaborative
36. King’s Clinical Trials Unit at King’s Health Partners
37. Manchester CTU (MCTU)
38. Oxford CTSU
39. Tayside CTU
40. OCTRU – Oxford Clinical Trials Research Unit
41. London School of Hygiene & Tropical Medicine CTU
42. Barts and The London Pragmatic CTU
43. NCTU, University of Nottingham
44. York Trials Unit
45. Birmingham CTU
46. Diabetes Trials Unit Oxford
47. Edinburgh CTU

Provisionally registered CTUs

1. Cambridge Epidemiology & Trials Unit
2. Exeter CTU
3. Hull Health trials unit
4. Lancashire CTU
5. Brighton and Sussex
