# Supplementary material for: A new trial monitoring plan (TMP) template for clinical trials: output from a Delphi process
Source: Trials. 2024 Nov 9;25:748. doi: 10.1186/s13063-024-08601-z (PMC11549859; doi:10.1186/s13063-024-08601-z)
Supplement: Supplementary file 3 — Supplementary Material 3: Additional items recommended by participants during Delphi round 1. [file 13063_2024_8601_MOESM3_ESM.docx]

**Supplementary file 3: Additional items recommended by participants during Delphi Round 1**

1. Space to store thresholds for metrics

2. Checks for unusual data patterns/Suspected fraud e.g., audit trail end digit review?

3. Have all SAEs been reported by site within the reporting timelines?

4. Review of consent forms to ensure completed correctly.

5. Consent - consider on-site activities to review consenting process and document completion.

6. Site selection/evaluation plan.
