# Supplementary material for: A new trial monitoring plan (TMP) template for clinical trials: output from a Delphi process
Source: Trials. 2024 Nov 9;25:748. doi: 10.1186/s13063-024-08601-z (PMC11549859; doi:10.1186/s13063-024-08601-z)
Supplement: Supplementary file 4 — Supplementary Material 4: Trial monitoring template. [file 13063_2024_8601_MOESM4_ESM.docx]

**<study name> Trial Monitoring Plan**

| **Trial Information** | |
| --- | --- |
| Trial Short Title/Acronym |  |
| Trial Long Title |  |
| ClinicalTrials.gov identifier |  |
| EudraCT number |  |
| ISRCTN number |  |

**This monitoring plan was created from the Trial Monitoring Plan (TMP) Template Version 2, dated 06Mar2024.**

| **Trial Monitoring Plan Information** | | | |
| --- | --- | --- | --- |
| **TMP Version** |  | | |
| **TMP Date** |  | | |
| **Reviewed by** | **Position** | **Signature** | **Date** |
|  |  |  |  |
|  |  |  |  |
| **Approved by** |  |  |  |
|  |  |  |  |
|  |  |  |  |

| **Summary of the changes made to the previous version** |
| --- |
|  |

**Introduction to this trial monitoring plan template for Clinical Trial Units (CTUs): [remove this page once the template is finalised]**

This document provides a monitoring plan template suitable for both CTIMP and non-CTIMP studies. The template includes sections on site initiation and close out visits, and various monitoring visits such as centralised, onsite, remote, routine and triggered. The template also includes sections on pharmacy, medical device, and sample monitoring visits.

Please tailor the template to align with your study's specific requirements and risk levels. Alternatively, you may choose to incorporate relevant sections into your trial monitoring plan. It’s essential to highlight that this template is not a standalone document and should be used in conjunction with the clinical trial protocol, CTU's study-specific SOPs, and any other work instructions specified by the CTU. Any template sections that do not pertain to the trial or CTU can be omitted as needed.

**Important note:** Throughout the template, you will find tables with a column called ‘Trial Specific Information’ which should be used for tailoring the template to the specific requirements of the trial. These tables also include examples and guidance for ‘Trial Specific Information’ column in italic font which you have the flexibility to choose whether to remove, retain, or enhance based on the trial's risk level or any specific regulations pertinent to the CTU. The template also includes checklists in various sections. Certain instructions are coloured in red text and should be deleted once the template is in its final form.

**List of Acronyms**

| **Acronyms** | **Definitions** |
| --- | --- |
| CRO | Contract Research Organization |
| CTIMP | Clinical Trial of an Investigational Medicinal Product |
| CTU | Clinical Trials Unit |
| DMEC | Data Monitoring and Ethics Committee |
| eCRF/CRF | Electronic/Case Report Form |
| FDA | Food and Drug Administration |
| GCP | Good Clinical Practice |
| IMP | Investigational Medicinal Product |
| ISF | Investigator Site File |
| non-CTIMP | Clinical Trial that does not involve an Investigational Medicinal Product |
| PSF | Pharmacy Site File |
| QA | Quality Assurance |
| SADE | Serious Adverse Device Effect |
| SAE | Serious Adverse Event |
| SDV | Source Data Verification |
| SmPC | Summary of Product Characteristics |
| SOP | Standard Operating Procedure |
| TMG | Trial Management Group |
| TMP | Trial Monitoring Plan |

**Table of Contents**

[**Purpose** 5](#_Toc160618410)

[**Study Details** 5](#_Toc160618411)

[**An overview of the trial design** 5](#_Toc160618412)

[**Summary of the trial risks** 6](#_Toc160618413)

[**Monitoring** 7](#_Toc160618414)

[**Site Initiation** 8](#_Toc160618415)

[**Centralised Monitoring Activities** 8](#_Toc160618416)

[**Centralised Quality Checks** 10](#_Toc160618417)

[**Onsite Monitoring Visit** 11](#_Toc160618418)

[**Remote Monitoring Visit** 14](#_Toc160618419)

[**Metrics** 15](#_Toc160618420)

[**Triggered Monitoring** 15](#_Toc160618421)

[**Pharmacy Monitoring** 16](#_Toc160618422)

[**Medical Device Monitoring** 17](#_Toc160618423)

[**Sample Monitoring** 17](#_Toc160618424)

[**Monitoring reports** 19](#_Toc160618425)

[**Site Close Out** 19](#_Toc160618426)

[**Sponsor Oversight** 20](#_Toc160618427)

[**External Vendor Oversight** 20](#_Toc160618428)

[**Archiving** 20](#_Toc160618429)

# **Purpose**

[This refers to the purpose of the TMP template. Enter a description of the purpose of this document based on the local SOPs and study protocol.]

*The Trial Monitoring Plan (TMP) template outlines a comprehensive set of planned and systematic measures designed to ensure that the <INSERT Study Name> is conducted, and data are created, recorded, and reported in accordance with Good Clinical Practice (GCP) and relevant regulatory standards. This plan is formulated based on the study-specific risk assessment and thus may be subject to modifications as the study risks evolve. Regular review of the TMP is advisable on an annual basis, with more frequent revisions warranted in the event of changes to the study's risk assessment or a protocol amendment.*

*The purpose of this template is to document the procedures for monitoring before, during and at the end of <Study Name> including centralised monitoring, site visits, report writing and archiving.*

# **Study Details**

Complete the table below to give a description of the study details.

|  | **Trial Specific Information** |
| --- | --- |
| CTIMP/non-CTIMP/Medical Device |  |
| Is there blinding in the study? |  |
| Who is blinded (give role) and what are they blinded to? |  |
| Randomisation procedure (for confirming that randomisation is performed according to the protocol and investigational plan) | *e.g., comparison of randomisation and CRF data to assess whether the subject was administered or dispensed the assigned product.* |
| Randomisation arms (if applicable) |  |
| Definition of end of trial |  |

# **An overview of the trial design**

[Complete the table below to give a description of the study design and trial overview.]

|  | **Trial Specific Information** |
| --- | --- |
| Study design |  |
| Primary Outcome measures |  |
| Describe any specific regulatory requirements: | *(e.g., The intervention is/is not being used in a licensed indication OR The data from the trial will/will not be used to support a licensing application OR The trial is/is not supporting a license change.*  *Include details of international regulation e.g., FDA, Medical Devices, or other specific regulations)* |
| Describe other issues specific to the treatment under study (anything that is not covered by inclusion/exclusion criteria). | *(e.g., participants need to fast for the intervention, or the treatment has a 12-hour life)* |

# **Summary of the trial risks**

The table below is a summary of the trial risk assessment. Please follow your local risk assessment procedures and complete any relevant documents accordingly. Please note this should not replace the risk assessment for the trial, instead, it should be used as a snapshot of the main risk assessment of the trial.

| Monitoring for this trial will be carried out using a risk-based approach. The risks to participants associated with the trial intervention(s) have been assessed in relation to standard care for the participant group concerned.  Is the monitoring of this trial carried out on a risk-based approach? Yes  No  For CTIMP trials (remove if a non CTIMP trial)  The trial has been assessed as:  A **Type A** trial requiring a **low** intensity of monitoring  A **Type B** trial requiring a **moderate** intensity of monitoring  A **Type C** trial requiring a **high** intensity of monitoring |
| --- |
| For CTIMP trials (remove if a non CTIMP trial)  Justification of risk category selected:  Add justification of risk category selected, *e.g., Impact of participation/administration of IMP compared to standard care, current licence vs. current off label use, oversight of IMP administration, side effects, safety monitoring.*  If a non-CTIMP trial, please indicate the trial risk level and justification of risk level: (remove if a CTIMP trial) |

# **Monitoring**

| The following monitoring approaches can be used based on the trial’s risk category and the trial specific risks identified within the risk assessment:  **Centralised monitoring** –Centralised monitoring is a remote evaluation carried out by sponsor personnel or representatives (e.g., clinical monitors, data management personnel, or statisticians) at a location other than the sites at which the clinical investigation is being conducted. Centralised monitoring processes can provide many of the capabilities of on-site monitoring as well as additional capabilities.  **On-site monitoring** – On-site monitoring will involve a visit to a site/s by a member of the CTU and can be carried out for the following reasons: Site Initiation Visit (SIV) (to train site staff), Triggered Monitoring Visit (TMV), a Standard monitoring visit (for example to conduct Source Data Verification (SDV) checks), and Close Out Visits (COV) (to close the site).  **Remote monitoring-** Remote monitoring is when monitors don’t visit the site to review the data. Instead, data monitoring is done virtually. With the use of digital technology, CROs and study sponsors/stakeholders can see the data from wherever they are located.  **The above list of monitoring approaches can be carried out as part of:**  **Triggered Monitoring-** Triggered monitoring in clinical trials is a risk-based monitoring approach where triggers specify the extent, timing, and frequency of monitoring visits.  **Routine Monitoring**- routine monitoring occurs at pre-decided times rather than in response to a concern.  **Important note:** All trial monitoring activities should be conducted in accordance with the local SOPs. The frequency of monitoring and who carries out the task should be indicated where possible for all monitoring tasks. | |
| --- | --- |
| Trial/Study Monitoring Approach: choose the monitoring approach for the trial. (Tick all that apply) | Centralised  Onsite  Remote |
| Type of monitoring (Tick all that apply) | Triggered  Routine |
| Frequency of routine monitoring visit? |  |
| Who will monitor the study? | *e.g., Sponsor/sponsor delegate* |
| What will be the first site monitoring time-point? | *e.g., after first participant is randomised, after IMP is shipped to site* |

# **Site Initiation**

The table below lists activities to be completed during the site initiation visit. Please complete this table considering the local SOPs and study protocol.

|  | **Trial Specific Information** |
| --- | --- |
| Remotely within the CTU or via on-site visits? Describe the rationale for the chosen method of Site Initiation. Where SIV is to be carried out via on-site visits, indicate how many sites are to be visited. | *(Format on-site visit/teleconference/video conference e.g., MS teams meeting).* |
| Determine site staff members who need to be present at the initiation visit. | *(e.g., principal investigator, investigator, study assistant, pharmacist, etc.).* |
| Attendance to be documented. | *list of attendees in person or online etc.* |
| List of trainings to occur during the site initiation. |  |
| Site-specific documentation reviews. | List all the documents to be checked.  *(e.g., Delegation logs, Training logs (including the trial-specific training), CVs and GCP certificates).* |
| Arrangements for study medication, documentation (CRFs, Investigator Site File) and further study material delivery to sites(s). | (*e.g., drug supply, schedule of assessments, treatment schedule, biological sample collection/ processing/ shipment*). |
| **Additionally, site initiation should include the following checklist:**   - If applicable, a review of the Pharmacy facilities (see pharmacy section of the template). - Request for submission of Trial Equipment calibration records. - Confirmation of Critical Documentation held both regulatory-specific, trial, and site-specific. | |

| **Centralised Monitoring Activities**  The table below lists activities to be completed during centralised monitoring. Please complete this table considering the local SOPs and trial protocol. |
| --- |
| \|  \| **Trial Specific Information** \| \| --- \| --- \| \| Frequency of centralised monitoring \|  \| \| **Consent and eligibility** \| \| \| Checking consent is taken correctly. \| *e.g., Completion of Informed Consent Forms (ICFs) for X participants (X is determined based on the trial risk), checking the signature, dates are contemporaneous, and counter signatory on the delegation log.* \| \| **Additionally, consent and eligibility should include the following checklist:**  (Add/Delete as appropriate to the trial)   - Eligibility checks before randomisation. - Review of trial eligibility criteria sign-off by local PI. \| \| \| **Site Delegation and training** \| \| \| Indicate how staff training and delegation of responsibilities will be monitored including the frequency of review. \| *(e.g., collection and review of CVs, training logs and delegation logs)* \| \| **IMP accountability**  **(For more details on IMP please see the pharmacy monitoring section in this TMP)** \| \| \| Will IMP accountability be done for all participants or for a sample from each site? \|  \| \| How will this be done if an external service provider is being utilised? \|  \| \| How frequently should the accountability log be sent to the trial office? \| *e.g., every 6 months* \| \| How frequently should the dispensing log be sent to the trial office? \| *e.g., every 6 months* \| \| **Additionally, IMP accountability should include the following checklist:**  (Add/Delete as appropriate to the trial)   - Review of IMP shipment and delivery documentation. - Review of IMP dosage calculations. \| \| \| **AE, SAE, and SADE** \| \| \| Adverse Events \| *e.g., centralised review or reconciliation of SAE forms and reporting timelines.* \| \| If medical device trial, indicate if SADEs are reported to the manufacturer or delegated to CTU. \|  \| \| Out-of-hours emergency cover arrangements- Where participants are provided with out of hours contact details for site staff. \| *(e.g., on a Participant ID card or PIS)* indicate how this will be verified *(e.g., for high-risk trials a test procedure may be put in place).* \| \| **Data Checks** \| \| \| Checks for missing or invalid data (range and consistency checks) \|  \| \| Checks for unusual data patterns/Suspected fraud. \| *e.g., audit trail end digit review*. \| \| Other- Specify (Add any other data checks to be done centrally) \|  \| \| **Protocol Deviation** \| \| \| Review of Visit Window Thresholds \|  \| \| Other- Specify (Add any protocol deviation which is to be checked centrally) \| *(*These examples are not an exhaustive list. *e.g., Centralised review of adherence to the time of randomisation and intervention consistent with protocol.)* \| \| **Site File Review** \| \| \| Site File and Pharmacy File: Indicate the procedures for checking the site file. \| *e.g., A checklist of documents contained within the site file and site pharmacy file will be sent out for self-completion by the sites as appropriate. The Trial Office will monitor these on return and implement remedial action as and when appropriate.* \| |

# **Centralised Quality Checks**

| **Centralised quality checks should include the following checklist:**  (Add/Delete as appropriate to the trial)   - Completion of a site questionnaire/assessments or to confirm that they can fulfil the safety requirements and perform the required trial assessments. - Sites are requested to return anonymised screening logs every X months *(X is determined based on the trial risk)* for review at CTU. - Chief Investigators and Principal Investigators sign an Investigator Statement agreeing to their roles and responsibilities during the trial. - Screening and Enrolment log review before each IDMC, or every X months *(X is determined based on the trial risk).* - Signed receipts to confirm receipt of any updated documents *(e.g., Protocols, Investigator Brochures (IBs) etc.)* as required. - Other centralised quality control procedures to be conducted centrally by trial team members [add as applicable]. |
| --- |

# **Onsite Monitoring Visit**

The table below lists activities to be completed at onsite monitoring visits. Please complete this table considering the local SOPs and study protocol.

|  | **Trial Specific Information** |
| --- | --- |
| Frequency of on-site monitoring |  |
| Choose the applicable option from the two following options and delete the one that is not needed:  On-site monitoring will be carried out for this trial in the form of routine monitoring visits with additional triggered monitoring visits where applicable OR  Routine on-site monitoring will not be carried out for this trial. Triggered monitoring visits will be conducted in response to the triggers stated in this trial monitoring plan or on request of the trial oversight committees, or the Senior Trial Manager/QA Manager at the CTU or the sponsor. |  |
| Availability of completed source documents and CRF for the monitoring visit. | *Indicate how this will be done prior to visit* |
| Selection criteria for participants to be reviewed during On-site Monitoring Visits- This may be on request of the TMG or following review of centralised monitoring reports. | (*e.g., participants who have a high number of SAEs reported* *or on a percentage of participants* *e.g., 10% selected at random*). |
| Review of consent forms to ensure completed appropriately. | *e.g., Completion of Informed Consent Forms (ICFs) for X participants (X is determined based on the trial risk), checking signature, dates are contemporaneous, and counter signatory on the delegation log.* |
| Serious Adverse Event / Serious Adverse Device Effect report(s) check:  Check all serious adverse events are accurately documented and reported by site within the reporting timelines. | *To be performed for <X> number /% of participants (X is determined based on the trial risk), at each monitoring visit alongside medical records and database entries/logs of SAEs.* |
| Review Medical/study records and results of eligibility assessments to confirm participant eligibility. | *for <X%> of participants (X is determined based on the trial risk).* |
| Investigator Site File - presence and completion of all (OR a selection of) trial documents, security, and location of files. | *List all the documents to be checked.*  *(e.g., Delegation logs, Training logs (including the trial-specific training), CVs and GCP certificates).* |
| Source document completion in accordance with the ALCOA principles check.  *ICH E6 4.9.0 -The investigator/institution should maintain adequate and accurate source documents and trial records that include all  pertinent observations on each of the site’s trial subjects. Source data should be attributable, legible, contemporaneous, original, accurate,  and complete. Changes to source data should be traceable, should not obscure the original entry, and should be explained if necessary (e.g.,  via an audit trail). |  |
| **Additionally, the onsite monitoring visit should include the following checklist:**  (Add/Delete as appropriate to the trial)   - Checking understanding and adherence to study protocol, procedures, and governance requirements (including any conditions in regulatory or ethics approval). - Verification that resources and facilities remain adequate. - Verification of appropriate oversight and documented delegation by the local investigator. | |
| **Protocol Deviation and Compliance** | |
| **Additionally, protocol deviation and compliance should include the following checklist:**  (Add/Delete as appropriate to the trial)   - Verification of missing visits, examinations, or tests. - Verification of lab reports reviewed, signed, and dated appropriately. - Verification of protocol deviations reported appropriately. - Verification of any new protocol deviations and/or regulatory or GCP deviations that occurred at the site since the last visit, reported appropriately. | |
| **Site staff discussion** | |
| **Site staff discussion should include the following checklist:**  (Add/Delete as appropriate to the trial)   - Discussions with site staff regarding staff training requirements (current documents and training present, staff changes documented, CVs, GCP, delegation log). - Time at the end of the monitoring visit for discussion with the site staff to resolve any issues where feasible. Key points to be recorded in the monitoring visit report. Issues not resolved during the visit should be recorded in the report for resolution prior to the next monitoring visit. | |
| **Documents and systems to be reviewed** | |
| Deviation logs | *e.g., frequency of checks* |
| Screening logs | *e.g., frequency of checks* |
| Completion of previously raised findings and actions (as appropriate). | *Indicate how this will be checked* |
| Randomisation processes | *e.g.,* *date of randomisation recorded on the CRF and randomisation service e.g., sealed envelope if applicable, and appropriateness of trial team member according to the site delegation log.* |
| Screening procedures |  |
| Early cessation of participation in trial (treatments, procedures, and/or data) |  |
| Hard-copy CRFs and patient completed questionnaires validation. | *The amount of content to be checked and the error rate (X) is determined based on the trial risk. Where the CRF is a hard copy, the content of approximately X% of case report forms (CRFs) and patient questionnaires entered at sites will be checked (or double entered) to ensure the accuracy of data input. An error rate of <X% will require no further action, however, if the error rate is >X%, a 100% check of forms will be undertaken.* |
| **Visit to other departments** | |
| Will the monitor visit the Lab, and Pharmacy? If yes, complete the relevant sections of this template. |  |
| **Source Data Verification (SDV)** | |
| Is any SDV to be performed? Yes/no |  |
| If applicable, which participants need SDV and how will you select them? | *e.g., number/percentage of participants and how you will select them, e.g. First X patient(s) at each site.* |
| If applicable, what data needs SDV? | *e.g., study arm, outcome data, or all data.* |
| Describe what source data will be available as a hard copy, and what will be available electronically and how access arrangements will be set up. *(*Refer to source data location agreement if applicable) |  |

##

# **Remote Monitoring Visit**

[The table below lists activities to be completed during remote monitoring visits. Please complete this table considering the local SOPs and study protocol.]

Remote Monitoring serves as a valuable resource for the CTU trial management team, allowing them to adopt a risk-based strategy for minimizing on-site monitoring and addressing situations where physical presence at the trial site is not feasible. It is crucial to prepare adequately for remote monitoring, ensuring that all necessary information and documentation are obtained from the trial site for the monitoring visit. This may include using self-monitoring questionnaires, administered in accordance with a risk-based approach, which the trial site team completes and submits to the CTU for confirmation. If requested documentation contains personal information, this should be redacted and managed accordingly i.e., no patient personal details will be retained by the CTU.

|  | **Trial Specific Information** |
| --- | --- |
| Frequency of remote monitoring. |  |
| Selection criteria for participants to be reviewed during Remote Monitoring Visits | *This may be on request of the TMG or following a review of centralised monitoring reports, (e.g., participants who have a high number of SAEs reported or on a percentage of participants e.g., 10% selected at random).* |
| Determine how the trial issue will be resolved. | *e.g., telephone, email* |
| *X should be determined based on the trial risk.*  **Monitoring will consist of X% monitoring of the following:** |  |
| Patient informed consent forms completed correctly. |  |
| CRF/eCRF completion and data cleaning |  |
| Recording and reporting of AEs and SAEs |  |
| Source data verification facility/all CRF entries can be verified either in electronic or paper format (see SDV section for more details). |  |
| **Monitoring will also consist of monitoring the following items as appropriate to the trial:** (Add/Delete as appropriate to the trial)  *Localised PIS and consent form*  *Delegation log (updated version to be sent if additional study team leave or join)*  *CV’s, GCP certificates and training logs*  *Monthly screening and recruitment logs (anonymised)*  *Deviation logs*  *eCRF audit logs*  *Local approval documents*  *Investigator Site File Contents (e.g., newsletters, significant communication with the site)*  *Pharmacy Site File Contents (see pharmacy section for more details).*  *IMP (request/shipment request, accountability logs, destruction logs, temperature logs).* | |

# **Metrics**

The table below is about the use of metrics in monitoring trials, sometimes termed “site performance metrics”. Add more rows as needed.

| **Metrics**  If applicable, list the metrics used for this trial. | **Thresholds**  If applicable, list the metrics thresholds for this trial. | **Frequency of metric checks** |
| --- | --- | --- |
|  |  |  |
| **Escalation** | | |
| Consider the action of escalation proportionate to centralised monitoring findings, linked to specific metrics crossing a threshold if appropriate. A general concern with a site based on correspondence or other incidents may also be a reason to escalate. Add action of escalation for trial in here i.e., when meetings will be held to discuss an action. | | |

# **Triggered Monitoring**

The table below is to be completed for triggered monitoring. Examples and guidance are given to help with developing trial triggered monitoring strategies. Please complete this table considering the local SOPs and study protocol. Please note this sections lays out the process leading to triggered monitoring. Once this is established, the monitoring will be done either centrally, on-site, or remote, in which the relevant sections should be completed for the visit.

| A *weekly/monthly/quarterly* triggered monitoring report (produced by the *Data Management team* OR *manually from the database by the study team*) will be generated to include data around specific trigger categories. For this trial, <xx> number of triggers in a <xx> (*X is determined based on the trial risk)* period will require further investigation by the TMG and, where necessary, may trigger an on-site visit. Triggered monitoring visits can also happen as a result of other events such as a phone call with the site. Any triggered monitoring visits will be documented internally within a monitoring report.  **Thresholds and associated actions/escalation plan:**  *(What specific findings/thresholds would necessitate an action in response? What would the immediate corrective actions be and what is the escalation process if the issue is not resolved within a specified timeframe?)*  **The types of issues identified through monitoring that would trigger immediate issue escalation:**  This is not an exhaustive list, items can be added or removed as appropriate.   - *A high level of findings through centralised monitoring oversight.* - *A high number of protocol deviations.* - *Low or High SAE reporting rate compared with other sites.* - *Poor data quality (long data entry delays, high query rate and high percentage of missing data, constant outstanding data, particularly relating to primary endpoint or safety data).* - *Concerns over IMP or sample management processes.* - *Concerns over consent procedures.* |
| --- |

# **Pharmacy Monitoring**

[The table below lists activities to be completed during pharmacy monitoring visits. Please complete this table considering the local SOPs and study protocol.]

|  | **Trial Specific Information** |
| --- | --- |
| Is Pharmacy involved in the study? Yes/No |  |
| **Planned Frequency of Pharmacy Monitoring Visits** | |
| Frequency of pharmacy monitoring visits? |  |
| Will Pharmacy visits be timed with the main visits? |  |
| **Ordering and Storage of IMP** | |
| Description of the necessary transport and storage conditions, if applicable with reference to the study protocol or another respective document. |  |
| **Additionally, ordering and storage of IMP should include the following checklist:**   - Checks to verify if IMP is being stored appropriately and in a secure location. - Checks to verify the availability of adequate stocks within expiry dates for the planned participants. - Checks to verify storage temperatures adequately monitored by pharmacy staff. - Checks for any temperature excursions. - Checks to verify any temperature excursions have been appropriately managed. | |
| **IMP Accountability** | |
| **IMP accountability should include the following checklist:**   - Checks to determine supplied IMP dispensed at the protocol-specified dose(s)/schedule. - Checks for any IMP returned or destroyed at the monitoring visit? (*e.g., expired, or damaged).* - Checks to verify the disposal of IMP at the site is appropriately documented. - Checks to verify study medication is appropriately documented. - Checks for any discrepancies in IMP accountability logs. | |
| **Pharmacy File** | |
| Pharmacy File: Indicate the documents to be checked for the pharmacy file. | *e.g., Is the current version of Investigator Brochure/SmPC, IMP handling/pharmacy guidelines (if applicable) and current approved protocol held in the Pharmacy File?* |

# **Medical Device Monitoring**

The table below lists activities to be completed during medical device monitoring. Please complete this table considering the local SOPs and study protocol.

|  | **Trial Specific Information** |
| --- | --- |
| Does this study involve Medical Devices? Yes/No |  |
| **Ordering and Storage of the devices** | |
| Description of the necessary transport and storage conditions, if applicable with reference to the study protocol or another respective document. |  |
| **Device Accountability** | |
| **Monitoring device accountability should include the following checklist:**   - Allocation/return - Storage - Expiry - Documentation | |

# **Sample Monitoring**

The table below lists activities to be completed during sample monitoring. Please complete this table considering the local SOPs and study protocol.

|  | **Trial Specific Information** |
| --- | --- |
| Does this study involve the collection of biological samples? Yes/No |  |
| Are the samples processed locally or centrally? |  |
| **Sample Monitoring Procedure** | |
| **Sample monitoring procedure should include the following checklist:**   - Sample log completion checks - Sample delivery log checks (if samples are sent off-site for processing). - Other documentation related to sampling. | |
| **Storage of samples** | |
| Description of the necessary transport and storage arrangements for samples collected for the study if applicable, with reference to the study protocol or another respective document. |  |
| How often will the labs be visited? |  |
| Where the lab section of the ISF will be held and who will maintain this? |  |
| Which procedures should be followed where deviations occur? |  |
| Procedures to take place during the close-out visit. |  |
| **Sample Accountability** | |
| **Sample accountability should include the following checklist:**   - Storage conditions - Sample tracking - Sample labels of trial identifiers - Laboratory reports - Expiry - Temperature logs | |

# **Monitoring reports**

Use this section to populate the monitoring report following a monitoring visit.

| The monitoring report is intended to summarise the monitoring visit. It will facilitate the recording of the items reviewed, any findings (such as non-compliance, deviations, deficiencies, or data anomalies), and recommended corrective actions. Furthermore, the report will enable the documentation of findings and details of any meetings conducted during the visit, which encompasses the feedback meeting. The full follow-up letter should include the following components: (this is not an exhaustive list)   - Actions Resolution Document - Date of visit - Name of the monitor(s) - Site name - Name of the investigator - A brief review of any additional meetings that took place, in particular the feedback meeting. - Sites response and implementation of corrective actions where appropriate.   **Additionally, timelines should be allocated for:**   - Internal review of the written report. - Written report submitted to sites (if applicable). - Sites response and implementation of corrective actions where appropriate. |
| --- |

# **Site Close Out**

The competent authority and research ethics committee should be notified within 90 days of the end of the trial. A summary report of the research is to be sent to the competent authority and research ethics committee within 12 months of the end of the trial. The funder is to be provided a final report at the end of the trial. An official close-out letter will be sent by the trial manager to each participating site once outstanding queries are finalised, and all data has been received.

|  | **Trial Specific Information** |
| --- | --- |
| Will visits be conducted remotely from CTU, on-site or centrally? |  |
| % and number of sites expected to be visited for Site Close-out. |  |
| Document how site close-out will be conducted for those sites that are not visited. | (*e.g., via teleconference, emails, and letters to sites*). |
| The proposed timing of Close-out visits. | *(X weeks from the end of the study).* |
| **Additionally, close out visit should include the following checklist:**   - The Trial Site File must be reviewed and confirmed as complete prior to archiving. - All outstanding payments must be reviewed and invoiced. - Drug accountability. - Ensure all SAEs are correctly reported including SDV if required. - Ensure all data queries are addressed and closed. - Final review of Investigator Site File (ISF)/Pharmacy Site File (PSF). | |

# **Sponsor Oversight**

This section is optional. Please remove if this is covered by the CTU’s audit SOPs.

| [sponsor name] is sponsor of the study and has delegated sponsor responsibilities to [CTU name] CTU, whose main roles and responsibilities are: (Remove or include additional responsibilities as per the Sponsor – [CTU name] CTU arrangement.)  If [sponsor name] is not the sponsor please adapt the above sentence. [remove this sentence if not necessary]   - The provision of trial management - Inclusion of a medical expert into the Trial Management Group (TMG) - The holder of the Trial Master File (TMF) - Regulatory document collection - Site selection and monitoring - Site contracts - CRF design and distribution - Resolution of site compliance and performance issues - Oversight of drug supply and management - Data management (for centralised monitoring) - The oversight of safety assessments & onward reporting to investigators and authorities - Database development and maintenance, including program for randomisation - Data analysis - Preparation of study report and manuscripts |
| --- |

# **External Vendor Oversight**

[For details on monitoring external vendors please follow the local SOPs and sponsor guidelines if necessary or remove this section otherwise.]

# **Archiving**

[Archiving plan for the trial should be drawn here following the local SOPs and sponsor guidelines.]
